# Long-term use of metformin and the molecular subtype in invasive breast carcinoma patients – a retrospective study of clinical and tumor characteristics

**DOI:** 10.1186/1471-2407-14-298

**Published:** 2014-04-28

**Authors:** Nikola Besic, Nika Satej, Ivica Ratosa, Andreja Gojkovic Horvat, Tanja Marinko, Barbara Gazic, Rok Petric

**Affiliations:** 1 Institute of Oncology Ljubljana, Zaloska 2, 1000 Ljubljana, Slovenia; 2 Community Health Centre Ljubljana, Krziceva 10, 1000 Ljubljana, Slovenia

**Keywords:** Breast carcinoma, Diabetes mellitus, Prognosis, Metformin

## Abstract

**Background:**

Metformin may exhibit inhibitory effects on cancer cells by inhibiting mTOR signaling pathway. The aim of our retrospective study was to examine if patients with breast carcinoma (BC) and diabetes mellitus (DM) receiving metformin have a lower stage of carcinoma in comparison to patients not receiving metformin, and if the use of metformin correlates with the molecular subtype of BC.

**Methods:**

A chart review of 253 patients with invasive BC and DM (128 on metformin and 125 not on metformin) was performed. Control group consisted of 320 consecutive patients with invasive BC without DM. BC subtypes were classified by immunohistochemical surrogates as luminal A (estrogen receptor [ER] + and/or progesterone receptor [PR]+, HER-2-), luminal B (ER + and/or PR+, HER-2+), HER-2 (ER-, PR-, HER-2+), triple-negative/basal (ER-, PR-, HER-2-).

**Results:**

Patients on metformin had a lower proportion of T3 or T4 tumors than patients who were not receiving metformin (16% vs. 26%; p = 0.035). No statistical difference was found between the two study groups in N stage. Patients with DM on metformin, with DM not on metformin and the control group had different molecular subtypes of BC (p = 0.01): the luminal A subtype was found in 78%, 83% and 71%, the luminal B in 12.6%, 9% and 11%, HER-2 in 0.8%, 1.6% and 8%, and the triple-negative/basal-like subtype in 8.6%, 6.4% and 10%, respectively.

**Conclusion:**

Our data indicate that long-term use of metformin use correlates with molecular subtype of BC in diabetics on metformin in comparison to diabetics not on metformin and patients without DM. However, most likely, different distribution of the molecular subtypes of BC in these three groups of patients was caused by other risk factors for breast carcinoma, such as age of patients or obesity.

## Background

Epidemiological studies show that patients with diabetes mellitus (DM) have an increased risk of breast carcinoma and that metformin treatment is associated with a reduction in cancer risk [1,2]. It is known that anti-diabetic drugs may have an impact on breast carcinoma [3,4]. Patients with type 2 diabetes exposed to sulphonylurea or exogenous insulin had a significantly increased risk of cancer-related mortality compared with patients exposed to metformin [5].

Jiralensung et al. [4] reported that diabetic patients with breast cancer receiving metformin and neoadjuvant chemotherapy have a higher pathological complete response rate than diabetics not receiving metformin. Although metformin treatment did not influence the overall survival in this retrospective study, these results have led to a huge interest in metformin as an anti-cancer agent [6]. Metformin may exhibit inhibitory effects on cancer cells by inhibiting the mTOR signaling pathway. Metformin has anti-proliferative effects in primary breast carcinoma (BC) tumors [7]. Metformin alone inhibits cell proliferation and induces apoptosis in different breast cancer cell lines (ERα-positive, HER2-positive, and triple-negative) [8]. Furthermore, metformin sensitizes breast cancer cells to the cytotoxic effect of chemotherapeutic drugs in vitro [8]. In BC patients without diabetes mellitus (DM), the gene set analysis revealed a reduced expression of p53, BRCA1 and cell cycle pathways after two-week of treatment with metformin [9]. Therefore, it is possible that metformin has also an impact on tumor extension and progression in breast carcinoma (BC) patients. The aim of our retrospective study was to examine if the patients with BC and DM receiving metformin have a lower stage of carcinoma when compared to patients not receiving metformin. Another aim was to find out whether long-term use of metformin correlates with the molecular subtype of BC.

## Methods

Altogether, 253 (median age 67; range 38–93 years) patients with DM were surgically treated for invasive breast carcinoma at a single comprehensive tertiary cancer center from 2005 to 2011. In the same department, around 800 BC surgical procedures are performed annually. Referral to our center has not changed over these years. In order to avoid selection bias, all 320 consecutive patients with BC without DM (median age 60, range 28–86 y.), who were surgically treated in our tertiary cancer comprehensive center in the first half of 2006 were included in our control group. A chart review of all 573 patients was 80 performed.

The following data on clinical and histopathological characteristics were collected: patients’ age, body mass index (BMI), TNM tumor stage, number of metastatic lymph nodes, presence of estrogen and progesterone receptors and HER-2 expression. Tumor stage, presence of regional metastases, distant metastases and residual tumor after surgery were assessed by TNM clinical classification system according to the UICC criteria from 2009 [10]. BMI was calculated as weight/height^2^ (kg/m^2^). Co-morbidity was assessed by the American Society of Anesthesiologists (ASA score) [11].

In this study, routine pathology reports of surgical specimens were used. Histological slides were examined by six pathologists experienced in breast pathology. The histological type of each tumor was defined according to the WHO classification system. Tumor grade was defined according to the modified Black’s nuclear grading system. Sentinel lymph nodes were examined by imprint cytology and immunohistochemistry in paraffin sections [12]. If sentinel nodes turned out to be tumor-free, no further axillary surgery was recommended. In case of metastasis in sentinel lymph nodes detected by imprint cytological investigation, the patient underwent axillary dissection during the same surgical procedure. In case of malignant involvement only in the paraffin section, axillary dissection was performed. For the purposes of this study, estrogen receptors (ER) and progesterone receptors (PR) were considered positive if 10% or more tumor cells showed positive staining. The status of HER-2 receptors was determined by immunohistochemistry and fluorescence in situ hybridization. HER-2–positive tumors were defined as 3+ receptor over-expression on IHC staining and/or gene amplification found on fluorescence in situ hybridization testing. Unfortunately, in the majority of our patients the expression of Ki-67 was not assessed, so we were not able to classify our patients according to the new St. Gallen Consensus 2013 [13] which defined the surrogate intrinsic subtypes of breast cancer according to ER, PR, HER-2 status and also Ki-67. In our study molecular subtypes of BC were classified by immunohistochemical surrogates as luminal A (ER + and/or PR+, HER-2-), luminal B (ER + and/or PR+, HER-2+), HER-2 (ER-, PR-, HER-2+), triple-negative/basal (ER-, PR-, HER-2-) as was done in the study of Wiechmann et al. from the Memorial Sloan-Kettering Cancer Center [14].

Factors recorded for this study included surgical breast cancer treatment (breast-conserving surgery vs. mastectomy), axillary surgery (sentinel lymph node biopsy vs. axillary dissection), adjuvant chemotherapy, hormonal treatment and/or treatment with trastuzumab.

Our study was reviewed and approved by the Institutional Review Board of the Institute of Oncology Ljubljana and was performed in accordance with the ethical standards laid down in an appropriate version of the 1964 Declaration of Helsinki. Our study was conducted with the understanding and the consent of the subjects. All our patients are asked during the first admission to our institute or during a follow-up visit to give a consent for study of her/his chart and/or bioptic material for scientific purposes. Since the Institutional Review Board of the Institute of Oncology Ljubljana approved this specific study, our patients were not asked to give a written consent on this specific study.

### Statistical analysis

Statistical analysis of these factors (comparison of metformin group vs. no metformin group and comparison of metformin group vs. no metformin group vs. control group) was performed by contingence tables, ANOVA for normally distributed numerical variables and non-parametric tests for non-normally distributed numerical variables. Multivariate logistic regression was done in order to find out which factors were predictive factors for presence of regional metastases. A p-value of 0.05 or less was considered statistically significant. For statistical analysis, SPSS 16.0 for Windows was used.

## Results

Median age of patients with diabetes, BMI, tumor size and number of metastatic lymph nodes was 67 years, 29.7 kg/cm^2^, 2.1 cm and 1, respectively. Characteristics of (1) patients treated with metformin, (2) patients not treated with metformin and (3) control group of patients are presented in Table 1. The tumor-specific therapy and outcome of all three groups of patients are presented in Table 2.

**Table 1:** Tumor and demographic characteristics of 253 patients with breast carcinoma and diabetes (128 on and 125 not on metformin) and 320 patients with breast carcinoma without diabetes

**Factor**	**Sub-group**	**Patients with breast carcinoma and diabetes without metformin**	**Patients with breast carcinoma and diabetes on metformin**	**Patients with breast carcinoma without diabetes**	**P1**	**P2**
**Median age (years)**		69	65	60	0.034	0.0001
**Median BMI (kg/m**^**2**^**)**		29.35	30.30	25.80	0.18	0.0001
**Median ASA score**		2	2	2	0.78	0.0001
**Median tumor size (cm)**		2.1	2.05	1.8	0.46	0.014
**Median number of metastatic lymph nodes**		0.5	1	0	0.79	0.78
**Age (years)**	70 or less	72	96	248	0.003	0.0001
	71 or more	53	32	72		
**BMI (kg/m**^**2**^**)**	Less than 30	70	58	258	0.122	0.0001
**(N = 562)**	30 or more	51	63	62		
**ASA score (N = 480)**	1	3	0	103	0.21	0.0001
	2	77	80	119		
	3	38	38	22		
**Diet only (N = 253)**	No	105	128	-	-	-
	Yes	20	0	-		
**Therapy with sulphonylurea**	No	78	69	320	-	-
	Yes	47	59	0		
**pT tumour stage**	pT1	57	63	192	0.031	0.0001
	pT2	35	45	87		
	pT3	6	9	20		
	pT4	27	11	21		
**T3 or T4 stage**	pT1 or pT2	93	108	279	0.035	0.003
	pT3 or pT4	33	20	41		
**N stage (N = 572)**	pN0	63	65	173	0.90	0.78
	pN1 or pN2	62	63	146		
**Number of metastatic lymph nodes (N = 572)**	0	63	63	173	0.68	0.75
	1–3	32	39	80		
	4 or more	30	26	66		
**M stage**	M0	120	124	313	0.75	0.55
	M1	5	4	7		
**Type of invasive carcinoma**	Ductal	103	115	274	0.086	0.23
	Lobular or other	22	13	46		
**Molecular subtype of carcinoma (N = 569)**	Luminal A	104	99	226	0.60	0.01
	Luminal B	11	16	35		
	HER-2	2	1	25		
	Triple negative	8	11	31		
**Tumor differentiation (N = 566)**	Well or moderate	69	73	177	0.67	0.89
	Poor	56	75	136		
**ER status (10% or more) (N = 570)**	Positive	113	115	258	0.97	0.008
	Negative	12	12	60		
**PR status (10% or more)**	Positive	96	90	207	0.28	0.049
	Negative	29	37	111		
**ER status (1% or more) (N = 570)**	Positive	114	116	263	0.87	0.011
	Negative	11	12	57		
**PR status (1% or more)**	Positive	106	99	218	0.13	0.001
	Negative	19	29	102		
**HER-2 (N = 569)**	Negative	112	110	257	0.46	0.06
	Positive	13	17	60		
**Triple-negative tumor (N = 569)**	No	117	116	286	0.49	0.53
	Yes	8	11	31		

P1: p-value (DM not on metformin vs. DM on metformin).

P2: p-value (DM not on metformin vs. DM on metformin vs. controls).

ER: estrogen receptor status.

PR: progesteron receptor.

**Table 2:** Carcinoma-related treatment in 253 patients with breast carcinoma and diabetes (128 receiving and 125 not receiving metformin) and 320 patients with breast carcinoma without diabetes

**Factor**	**Sub-group**	**Patients with breast carcinoma and diabetes without metformin (N = 125)**	**Patients with breast carcinoma and diabetes on metformin (N = 128)**	**Patients with breast carcinoma without diabetes (N = 320)**	**P1**	**P2**
**Breast surgical procedure**	Quadrantectomy or lumpectomy	48	59	157	0.21	0.13
	Mastectomy	77	69	163		
**Axillary surgical procedure**	Sentinel node biopsy	54	61	147	0.43	0.73
	Lymphadenectomy	71	67	173		
**Adjuvant chemotherapy**	No	94	87	196	0.20	0.02
	Yes	31	41	124		
**Adjuvant hormone therapy**	No	14	15	76	0.89	0.0001
	Yes	111	113	244		
**Adjuvant trastuzumab**	No	118	115	285	0.17	0.22
	Yes	7	13	35		
**Adjuvant radiotherapy**	No	60	52	135	0.23	0.44
	Yes	65	76	185		

P1: p-value (DM not on metformin vs. DM on metformin).

P2: p-value (DM not on metformin vs. DM on metformin vs. controls).

Patients with DM were older than patients without DM (p < 0.001), had a larger median BMI (29.7 vs. 25.8; p = 0.0001), a larger median tumor diameter (2.1 vs. 1.8 cm; p = 0.004) and a higher tumor stage (T1/T2: 79% vs. 87%; T3/T4: 21% vs. 13%; p = 0.01). Patients with DM, as compared to patients without DM, showed no statistical difference in the rate of regional (50% vs. 47%) or distant metastases (3.6% vs. 2%) or in the median number of metastatic lymph nodes (1 vs. 0), respectively. Tumors in patients with DM were more often positive for ER (90% vs. 81%) and PR (74% vs. 65%) than tumors in patients without DM (p < 0.03). So, patients with DM were more often treated with hormones and less often with chemotherapy than patients without DM (p < 0.01). Tumors were HER-2 positive in patients with and without DM in 12% and 19% (p = 0.03), respectively. Patients with DM and the control group had different molecular subtypes of BC (p = 0.01): the luminal A subtype was found in 80% and 71%, the luminal B in 11% and 11%, HER-2 in 1% and 8%, and the triple-negative/basal-like subtype in 7% and 10%, respectively.

DM type 1 and DM type 2 were present in 40 and 213 cases, respectively. Altogether, 128 patients (median age 65; range 39–88 years) were on metformin, while 125 (median age 69; range 37–93 years) were not. Compared to patients not receiving metformin, a larger proportion of patients on metformin were younger than 71 years (p = 0.003) and had a smaller T stage (T1: 49% vs. 46%; T2: 35% vs. 28%; T3: 7% vs. 5%; T4: 9% vs. 21%, p = 0.03). Patients on metformin had a lower proportion of T3 or T4 tumors than patients who were not receiving metformin (16% vs. 26%; p = 0.035). No statistical difference was found between the two study groups in N stage (p = 0.90). Median tumor size (2.05 cm vs. 2.1 cm; p = 0.46), tumor grade, median number of metastatic lymph nodes (1 vs. 0.5; p = 0.79), ER status (p = 0.97), PR status (p = 0.28), HER-2 status (p = 0.46) or molecular subtypes of BC (p = 0.60) did not show any statistical difference between the two study groups (Table 1). There was a trend for a higher rate of ductal type of BC in patients with DM on metformin in comparison to those not receiving metformin (90% vs. 82%, p = 0.086). There was no statistical difference in the rate of lymphadenectomy or treatment with radiotherapy, chemotherapy, hormonal therapy or trastuzumab between the two groups of patients with DM. Patients with DM on metformin, those with DM not on metformin and the control group had different molecular subtypes of BC (p = 0.01): the luminal A subtype was found in 78%, 83% and 71%, the luminal B in 12.6%, 9% and 11%, HER-2 in 0.8%, 1.6% and 8%, and the triple-negative/basal-like subtype in 8.6%, 6.4% and 10%, respectively.

Age, BMI, hormone receptor status, HER2 status, tumor grade and molecular subtype were included in the multivariate analysis in order to find out which were independent predictive factors for the presence of regional metastases. Only a tumor differentiation was independent predictive factor for the presence of regional metastases.

## Discussion

The aim of our study was to find out if the patients with BC and DM receiving metformin have a lower stage of carcinoma when compared to patients not receiving metformin. Our hypothesis was that the use of metformin slows down the progression of breast carcinoma in comparison to other types of anti-diabetic drugs. We found that patients on metformin had a lower proportion of T3 or T4 tumors than patients who were not receiving metformin (16% vs. 26%; p = 0.035). However, there was no significant difference in tumor diameter, tumor grade or median number of metastatic lymph nodes between the two study groups. Our patients using metformin had the same rate of ER and PR as those not receiving metformin. Thus, our data do not confirm the findings of Berstein et al. [15] who, in 90 postmenopausal BC patients with DM, observed a higher rate of positive progesterone receptors in patients on metformin when compared to those on sulphonylurea or insulin (73% vs. 37%).

Aksoy S et al. investigated the demographic and clinico-pathological characteristics of metformin users in comparison with patients without diabetes matched with the same age at the time of breast cancer diagnosis [16]. Patients who received insulin treatment were excluded. Metformin users had lower incidence of grade 3 tumors and lower incidence of triple-negative disease [16]. On the other hand, hormone receptor positivity was significantly higher in metformin users compared to nonusers; thus, hormonal treatment history was higher in metformin users [16]. Our patients using metformin did not have lower incidence of grade 3 tumors or lower incidence of triple-negative disease in comparison to diabetics not on metformin and/or patients without DM. But hormone receptor positivity was higher in our metformin users, so more metformin users had hormonal treatment in comparison to nonusers or patients without DM.

There is an emerging body of evidence supporting the hypothesis that short-term use of metformin has an impact on BC tumor cells in newly diagnosed, untreated, non-diabetic early-stage breast cancer patients [2,7,9,17]. Ki67 staining in invasive tumor tissue decreased in surgical specimen in patients who received metformin after diagnostic core biopsy [7]. A similar study was conducted by Hadad et al. [9] who observed a reduced expression of p53, BRCA1 and cell cycle pathways after 2-week treatment with metformin in BC patients without DM [9]. However, we were not interested in short-term action of metformin use. The aim of our study was to find out if long-term use of metformin correlates with the molecular subtypes of BC. We found that patients with DM on metformin, those with DM not on metformin and the control group of patients without DM had different molecular subtypes of BC: the luminal A subtype was found in 78%, 83% and 71%, the luminal B in 12.6%, 9% and 11%, HER-2 in 0.8%, 1.6% and 8%, and the triple-negative/basal-like subtype in 8.6%, 6.4% and 10%, respectively. However, the comparison of the molecular subtypes in a group of patients with DM on metformin and in those not receiving metformin did not show statistically different distribution. Thus, our data do not support the hypothesis that long-term use of metformin in diabetics correlates with the distribution of the molecular subtype of BC. Most likely, different distribution of the molecular subtypes of BC in these three groups of patients was caused by other risk factors for breast carcinoma, such as age of patients or obesity.

Xiao et al. [18], studied a clinical-pathological characteristic in Luminal A subtype of breast cancer, Luminal B (high Ki67) and Luminal B (Her-2+) subtype. They found out that luminal subtype was present in 68% of patients with BC and 10% of them had DM. They reported data about 1,384 Luminal A-subtype breast cancer patients, including 201 patients with diabetes; 3, 393 Luminal B (high Ki67)-subtype breast cancer patients, including 341 patients with diabetes; and 1,008 Luminal B (Her-2+)-subtype breast cancer patients, including 138 patients with diabetes [18]. A Cox multivariate regression analysis showed that among Luminal A and Luminal B (Her-2+) subtype patients, the metformin group had a better prognosis than did the non-metformin group, but there was no difference in prognosis between the metformin group and the non-diabetic group. For the Luminal B (high Ki67) subtype, the metformin group had a better prognosis than both the non-metformin group and the non-diabetic group [18].

Bayractar et al. [19] studied whether the use of metformin during adjuvant chemotherapy has an impact on the survival of patients with triple-negative BC. The study cohort was comprised of 63 diabetic patients receiving treatment with metformin, 67 diabetic patients not receiving metformin, and 1318 non-diabetic patients [19]. They found that metformin use during adjuvant chemotherapy did not affect the survival outcomes in diabetic patients with triple-negative breast cancer [19]. In our diabetic patients, as compared to those without DM, the rate of triple-negative BC was not significantly different. Metformin use in our diabetic patients was not correlated with the presence of triple-negative BC. The rate of triple-negative BC in our patients with DM on metformin, those not on metformin and controls was 8.7%, 6.4% and 9.7%, respectively.

There are several limitations of our study. It is retrospective, observational and non-randomized. Besides, data about the length of treatment with anti-diabetic drugs are missing. Furthermore, our patients received different combinations of anti-diabetic drugs and insulin types and doses. Yet, despite the fact that both DM and breast carcinoma are common diseases, the data about histopathological characteristics and the extent of the disease in these patients in the literature are scarce and conflicting [4,15,16,18-22]. Wolf et al. [20] found that BMI, tumor size and stage were larger among diabetic patients, while N or M tumor stage did not differ among patients with and without DM. They found that a more advanced stage in patients with DM could not be attributed to parity, family history of breast cancer, obesity, or other risk factors for breast cancer [20,23]. Similarly, our patients with DM were older, had a higher BMI, ASA score, mean tumor diameter and also a higher rate of T3/T4 tumors compared to the control group. Furthermore, in our patients with DM, there was no statistical difference in the rate of regional metastases or in the median number of metastatic lymph nodes when compared to patients without DM.

## Conclusion

Patients with DM have locally more advanced disease but do not have more advanced regional or distant disease when compared to patients without DM. Our data show that long-term use of metformin in diabetics is correlated with a lower local tumor stage and is not correlated with regional or distant disease. In addition, our data indicate that long-term use of metformin use correlates with molecular subtype of BC in diabetics on metformin in comparison to diabetics not on metformin and patients without DM. However, most likely, different distribution of the molecular subtypes of BC in these three groups of patients was caused by other risk factors for breast carcinoma, such as age of patients or obesity.

## Competing interests

Authors declare that there is no conflict of interest that could be perceived as prejudicing the impartiality of this paper.

## Authors’ contributions

NB participated in the design of the study, partially collected data and performed the statistical analysis. NS participated in collecting data and drafted the manuscript. IR, AGH, TM, BG and RP partially collected data. All authors read and approved the final manuscript.

## Pre-publication history

The pre-publication history for this paper can be accessed here:

http://www.biomedcentral.com/1471-2407/14/298/prepub
